# Statin-induced apoptosis via the suppression of ERK1/2 and Akt activation by inhibition of the geranylgeranyl-pyrophosphate biosynthesis in glioblastoma

**DOI:** 10.1186/1756-9966-30-74

**Published:** 2011-08-10

**Authors:** Masashi Yanae, Masanobu Tsubaki, Takao Satou, Tatsuki Itoh, Motohiro Imano, Yuzuru Yamazoe, Shozo Nishida

**Affiliations:** 1 Division of Pharmacotherapy, Kinki University School of Pharmacy, Kowakae, Higashi-Osaka 577-8502, Japan; 2 Department of Pharmacy, Sakai Hospital, Kinki University School of Medicine, Sakai, Osaka 590-0132, Japan; 3 Department of Pathology, Kinki University School of Medicine, Osakasayama, Osaka 589-8511, Japan; 4 Department of Surgery, Kinki University School of Medicine, Osakasayama, Osaka 589-8511, Japan; 5 Department of Pharmacy, Kinki University Hospital, Osakasayama, Osaka 589-8511, Japan

**Keywords:** statins, C6 glioma, ERK, Akt

## Abstract

**Background:**

Statins are inhibitors of 3-hydroxy-3-methylglutaryl-coenzyme A reductase, the rate-limiting enzyme in cholesterol synthesis. The inhibition of this key enzyme in the mevalonate pathway leads to suppression of cell proliferation and induction of apoptosis. However, the molecular mechanism of apoptosis induction by statins is not well understood in glioblastoma. In the present study, we attempted to elucidate the mechanism by which statins induce apoptosis in C6 glioma cells.

**Methods:**

The cytotoxicity of statins toward the C6 glioma cells were evaluated using a cell viability assay. The enzyme activity of caspase-3 was determined using activity assay kits. The effects of statins on signal transduction molecules were determined by western blot analyses.

**Results:**

We found that statins inhibited cell proliferation and induced apoptosis in these cells. We also observed an increase in caspase-3 activity. The apoptosis induced by statins was not inhibited by the addition of farnesyl pyrophosphate, squalene, ubiquinone, and isopentenyladenine, but by geranylgeranyl-pyrophosphate (GGPP). Furthermore, statins decreased the levels of phosphorylated extracellular signal-regulated kinase 1/2 (ERK1/2) and Akt.

**Conclusions:**

These results suggest that statins induce apoptosis when GGPP biosynthesis is inhibited and consequently decreases the level of phosphorylated ERK1/2 and Akt. The results of this study also indicate that statins could be used as anticancer agents in glioblastoma.

## Background

Glioblastoma is the most common type of malignant brain tumor and its prognosis is very poor. Surgical resection and chemotherapy are common treatments [1]. Despite recent advances in the understanding of the molecular mechanism of tumorigenesis, the outcome of malignant glioma remains poor [2]. Thus, it is imperative that new effective forms of therapy are developed for its treatment.

Statins are cholesterol-lowering agents that inhibit 3-hydroxy-3-methylglutaryl-coenzyme A (HMG-CoA) reductase, which catalyzes the conversion of HMG-CoA into mevalonate. Mevalonate is converted into farnesyl pyrophosphate (FPP) or geranylgeranyl pyrophosphate (GGPP) that can be anchored onto intracellular proteins through prenylation, thereby ensuring the relocalization of the target proteins in the cell membranes [3-5]. Inhibition of HMG-CoA reductase results in alteration of the prenylation of small G proteins such as Ras, which regulates cell growth and survival via the downstream signaling pathways [3-5]. Accordingly, inhibition of HMG-CoA reductase by statins was found to trigger apoptosis in several cancer cells [3-5]. We recently showed that statins decreased the activation of the Ras/extracellular regulated kinase 1/2 (ERK1/2) pathway and Ras/phosphoinositol-3 kinase/Akt pathway [3,4]. In malignant glioma cells, statins induce apoptosis by the activation of c-Jun N-terminal kinase 1/2 (JNK1/2) or by increasing the expression of Bim [6,7]. However, several aspects of the mechanism by which statins induce apoptosis in glioma cells remain unclear. In the present study, we investigated the mechanism by which statins induce apoptosis in rat C6 glioma cells.

## Materials and methods

### Materials

Mevastatin was purchased from Sigma (St. Louis, MO, USA), fluvastatin from Calbiochem (San Diego, CA, USA), and simvastatin from Wako (Osaka, Japan). These reagents were dissolved in dimethyl sulfoxide (DMSO) and filtered through syringe filters (0.45 μm; Iwaki Glass, Tokyo, Japan). The dissolved reagents were resuspended in phosphate-buffered saline (PBS, pH 7.4) and used in the various assays described below.

Mevalonic acid lactone (MVA), FPP, GGPP, squalene, ubiquinone, isopentenyladenine, and dolichol were purchased from Sigma. These reagents were dissolved in DMSO. These dissolved reagents were then resuspended in PBS (0.05 M; pH 7.4) and filtered through syringe filters (0.45 μm; Iwaki Glass) before use.

### Cell culture

C6 glioma cells were supplied by Dr. Takashi Masuko (Kinki University, Osaka, Japan) and cultured in Dulbecco's Modified Eagle's Medium (Sigma) supplemented with 10% fetal calf serum (FCS) (Gibco, Carlsbad, CA, USA), 100 μg/ml penicillin (Gibco), 100 U/ml streptomycin (Gibco), and 25 mM HEPES (pH 7.4; Wako) in an atmosphere containing 5% CO_2_. U251MG cells were provided by Health Science Research Resources Bank (Osaka, Japan) and cultured in minimum essential medium (Sigma) supplemented with 10% fetal calf serum (Gibco), 100 μg/ml penicillin (Gibco), 100 U/ml streptomycin (Gibco), and 25 mM HEPES (pH 7.4; Wako) in an atmosphere containing 5% CO_2_.

### Cell viability

Cell viability was quantified by using a trypan blue dye assay. The cells (2000 cells/well) were plated in 96-well plates and incubated with various concentrations of mevastatin, fluvastatin, and simvastatin for 24, 48, and 72 h. After incubation, the cells were stained with trypan blue, and the number of stained cells was counted.

### Measurement of caspase-3 proteolytic activity

We measured the caspase-3-like enzyme activity by monitoring proteolytic cleavage of the fluorogenic substrate Asp-Glu-Val-Asp-7-Amino-4-trifluoromethylcoumarin (DEVD-AFC) using the ApoTarget caspase-3 protease assay kit (BioSource International Inc., Camarillo, CA). The C6 glioma cells were incubated with or without mevastatin, fluvastatin, and simvastatin for 24 h. The cells were then collected, washed in PBS, and lysed in the lysis buffer provided in the aforementioned kit. The assay was performed by incubating a solution of cell lysates containing a 50 μM substrate at 37°C for 1 h. We fluorometrically measured the release of 7-amino-4-methylcoumarin from the substrate by using a fluorescence spectrophotometer (F-4010, Hitachi) at an emission wavelength of 505 nm and an excitation wavelength of 400 nm. Caspase-3 activity (measured on the basis of proteolytic cleavage of the caspase-3 substrate DEVD-AFC) was expressed in terms of change in substrate concentration (in pM) per h per mg of protein, after correction for the protein content of the lysates; the protein content of the cell lysate was determined by using the bicinchoninic acid (BCA) protein assay kit (Pierce, Rockford, IL, USA).

### Western blotting

C6 glioma cells treated with statins were lysed with a lysis buffer containing 20 mM Tris-HCl (pH 8.0), 150 mM NaCl, 2 mM EDTA, 100 mM NaF, 1% NP-40, 1 μg/ml leupeptin, 1 μg/ml antipain, and 1 mM phenylmethylsulfonyl fluoride. The protein content in the cell lysates was determined using a BCA protein-assay kit. The extracts (40 μg protein) were fractionated on polyacrylamide-SDS gels and transferred to polyvinylidene difluoride (PVDF) membranes (Amersham, Arlington Heights, IL, USA). The membranes were blocked with a solution containing 3% skim milk and incubated overnight at 4°C with each of the following antibodies: anti-phospho-ERK1/2 (Thr202/Tyr204), anti-phospho-Akt (Ser473), anti-phospho-JNK1/2 (Thr183/Tyr185), anti-ERK1/2, anti-Akt, and anti-JNK1/2 (Cell Signaling Technology, Beverly, MA, USA). Subsequently, the membranes were incubated for 1 h at room temperature with horseradish peroxidase-coupled anti-rabbit IgG sheep antibodies (Amersham). The reactive proteins were visualized using ECL-plus (Amersham) according to the manufacturer's instructions.

### Statistical analysis

All results are expressed as mean ± SD of several independent experiments. Multiple comparisons of the data were performed by analysis of variance (ANOVA) with Dunnett's test. *P *values less than 5% were regarded as significant.

## Results

### Effects of statins on C6 glioma cell proliferation and viability

**Figure 1:**
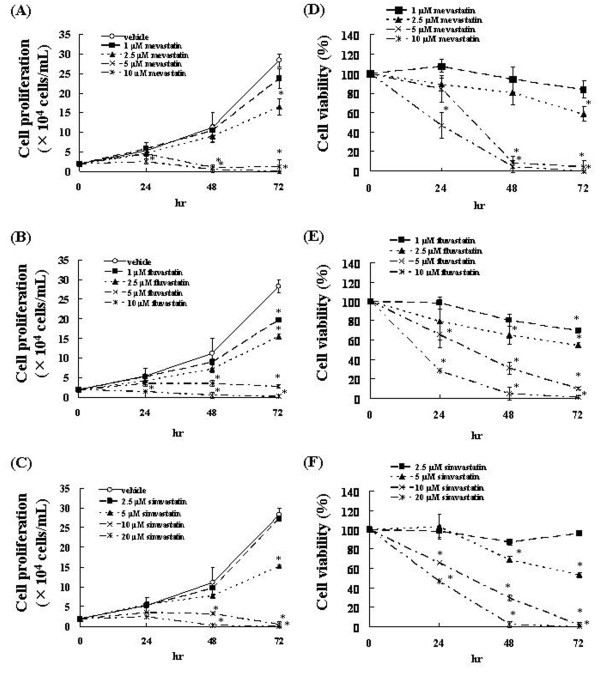
**Effects of statins on C6 glioma cell proliferation and viability**. (A-C) C6 glioma cells were incubated at a concentration of 2 × 10^4 ^cells/ml for 24 h in a 96-well plate. These cells were treated with various concentrations of statins. After incubation for 24, 48, or 72 h, the number of viable cells was counted by trypan blue staining. The results are representative of 5 independent experiments. *p < 0.01 vs. controls (ANOVA with Dunnett's test). (D-F) C6 glioma cells were treated with various concentrations of statins and trypan blue exclusion test was performed after 24, 48, or 72 h. The results are representative of 5 independent experiments. *p < 0.01 vs. controls (ANOVA with Dunnett's test).

To examine the cytotoxic effects of mevastatin, fluvastatin, or simvastatin on C6 glioma cells, C6 glioma cell proliferation was assessed in the presence of mevastatin (1-10 μM), fluvastatin (1-10 μM), or simvastatin (2.5-20 μM). We found that statins inhibited the C6 glioma cell proliferation in a concentration- and time-dependent manner (Figure 1A-C).

We also determined the cell survival rate, which was defined as the number of living cells at 24, 48, and 72 h after exposure to these agents at various concentrations compared with the number of live control (0.1% DMSO-treated) cells. The survival rates on exposure to 1, 2.5, 5, and 10 μM of mevastatin were 83.82%, 58.23%, 4.41%, and 0.52%, respectively, at 72 h (Figure 1D). Thus, the number of C6 glioma cells significantly decreased at 72 h after the administration of 5 and 10 μM mevastatin. The survival rates on exposure to 1, 2.5, 5, and 10 μM of fluvastatin were 69.70%, 54.71%, 9.71%, and 0.88%, respectively, at 72 h (Figure 1E). Thus, the number of C6 glioma cells significantly decreased at 72 h after the administration of 5 and 10 μM fluvastatin. The survival rates on exposure to 2.5, 5, 10, and 20 μM of simvastatin were 96.17%, 53.82%, 1.76%, and 0.49%, respectively, at 72 h (Figure 1F). Thus, the number of C6 glioma cells significantly decreased at 72 h after the administration of 10 and 20 μM simvastatin. On the basis of these results, 5, 5, and 10 μM were determined to be the cytotoxic concentrations of mevastatin, fluvastatin, and simvastatin, respectively.

**Figure 2:**
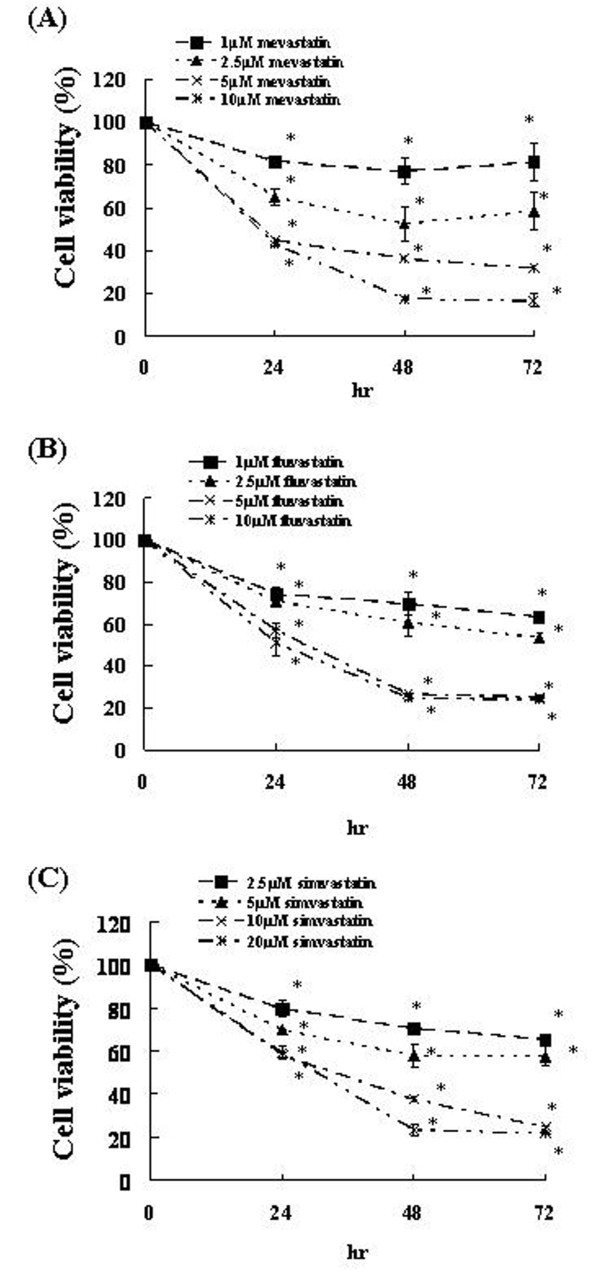
**Effects of statins on U251MG cell viability**. U251MG cells were treated with various concentrations of statins and trypan blue exclusion test was performed after 24, 48, or 72 h. The results are representative of 5 independent experiments. *p < 0.01 vs. controls (ANOVA with Dunnett's test).

To examine the cytotoxic effects of mevastatin, fluvastatin, or simvastatin on U251MG cells, the survival of these cells was assessed in the presence of mevastatin (1-10 μM), fluvastatin (1-10 μM), or simvastatin (2.5-20 μM). We determined the cell survival rate, which was defined as the ratio of the number of living cells after 24, 48, and 72 h of incubation with 1, 2.5, 5, 10 μM mevastatin, 1, 2.5, 5, and 10 μM fluvastatin or 2.5, 5, 10, and 20 μM simvastatin to the number of living cells in the control (0.1% DMSO-treated) samples. The survival rates on exposure to 1, 2.5, 5, and 10 μM of mevastatin were 81.44%, 58.41%, 31.81%, and 16.93%, respectively, at 72 h (Figure 2A). Thus, the number of U251MG cells significantly decreased at 72 h after the administration of 5 and 10 μM mevastatin. The survival rates on exposure to 1, 2.5, 5, and 10 μM of fluvastatin were 63.37%, 53.71%, 25.45%, and 24.08%, respectively, at 72 h (Figure 2B). Thus, the number of U251MG cells significantly decreased at 72 h after the administration of 5 and 10 μM fluvastatin. The survival rates on exposure to 2.5, 5, 10, and 20 μM of simvastatin were 65.57%, 57.59%, 25.11%, and 21.87%, respectively, at 72 h (Figure 2C). Thus, the number of U251MG cells significantly decreased at 72 h after the administration of 10 and 20 μM simvastatin.

### Statins-mediated activation of caspase-3

**Figure 3:**
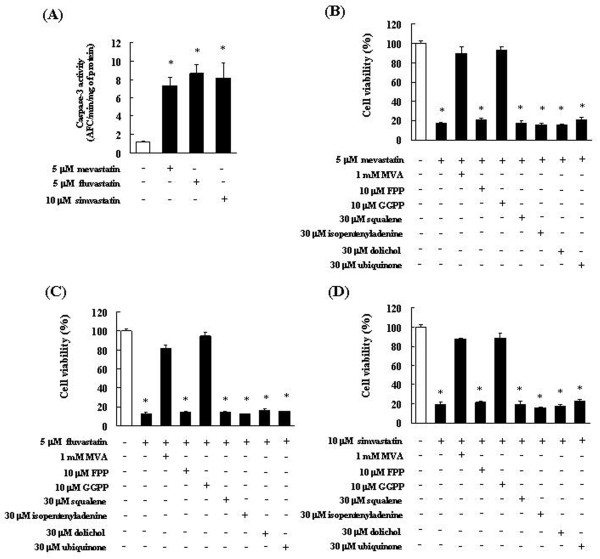
**Inhibition of statin-induced apoptosis in C6 glioma cells by intermediates of the mevalonate pathway**. (A) Induction of caspase-3-like activity associated with statin-induced cell death. Caspase-3 activity is expressed as pM of proteolytic cleavage of the caspase-3 substrate Asp-Glu-Val-Asp-7-Amino-4-trifluoromethylcoumarin (DEVD-AFC) per h per mg of protein. The results are representative of 5 independent experiments. *p < 0.01 vs. controls (ANOVA with Dunnett's test). (B-D) C6 glioma cells were pretreated with 1 mM mevalonic acid lactone (MVA), 10 μM farnesyl pyrophosphate (FPP), 10 μM geranylgeranyl pyrophosphate (GGPP), 30 μM squalene, 30 μM isopentenyladenine, 30 μM ubiquinone, or 30 μM dolichol for 4 h and then treated with (B) 5 μM mevastatin, (C) 5 μM fluvastatin, or (D) 10 μM simvastatin for 72 h. These results are representative of 5 independent experiments. *p < 0.01 vs. the controls (ANOVA with Dunnett's test).

The cytotoxic effects of statins on C6 glioma cells were attributed to the induction of apoptosis, as demonstrated by the results of the following biochemical assays. We investigated the involvement of statins in caspase-3 activation. Caspase-3 activity was measured at 24 h after the addition of 5 μM mevastatin, 5 μM fluvastatin, 10 μM simvastatin to the C6 glioma cells. We observed that the addition of statins resulted in a marked increase in caspase-3 activity in comparison with that in the control (0.1% DMSO-treated cells) (Figure 3A).

### Combined effects of intermediate in the mevalonate pathway on the apoptosis-inducing effect of statins

To study the combined effects of MVA, FPP, GGPP, squalene, isopentenyladenine, dolichol, and ubiquinone on the apoptosis-inducing effect of statins, C6 glioma cells were pre-administered 1 mM MVA, 10 μM FPP, 10 μM GGPP, 300 μM squalene, 30 μM isopentenyladenine, 30 μM dolichol, and 30 μM ubiquinone. Mevastatin, fluvastatin, or simvastatin were added to cell suspensions to a concentration of 5, 5, or 10 μM. After 72 h, the cell viability was measured by the trypan blue dye method described above. The statins did not show any significant difference in cell viability in the presence of FPP, squalene, isopentenyladenine, dolichol, and ubiquinone. However, pretreatment with MVA and GGPP caused the statin-induced apoptosis to be significantly inhibited (Figure 3B-D).

### Statin-induced decrease in the expressions of phosphorylated ERK1/2 and Akt

**Figure 4:**
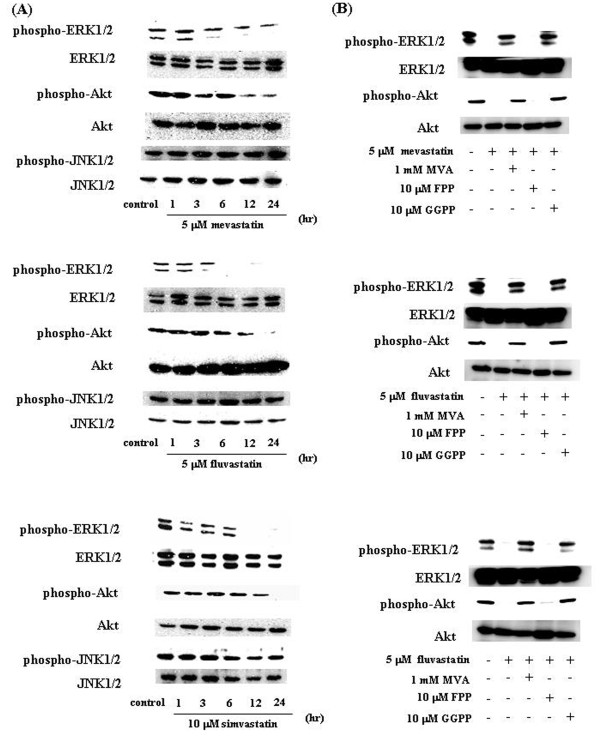
**Statins specifically suppress the activation of Ras/extracellular signal-regulated kinase (ERK) and Ras/Akt pathways in C6 glioma cells**. (A) C6 glioma cells were treated with 5 μM mevastatin, 5 μM fluvastatin, or 10 μM simvastatin for 1, 3, 6, 12, or 24 h. Control cells were treated with 0.1% DMSO and cultured in serum-containing medium for 24 h. Whole-cell lysates were generated and immunoblotted with antibodies against phosphorylated ERK1/2 (phospho-ERK1/2), phosphorylated Akt (phospho-Akt), phosphorylated c-Jun N-terminal kinase 1/2 (phospho-JNK1/2), ERK1/2, Akt, and JNK1/2. (B) ERK1/2 and Akt activation in C6 cells to which statins were administered with or without the addition of MVA, FPP, and GGPP. Phospho-ERK1/2, phospho-Akt, ERK1/2, and Akt levels were determined by immunoblotting analysis of the whole-cell lysate.

To identify the molecules involved in statin-induced apoptosis, we investigated the Ras downstream cascade that statins may inhibit in order to induce apoptosis. Statins inhibited the expression of phosphorylated ERK1/2 and Akt, as downstream Ras. There was no substantial change in the level of phosphorylated JNK1/2 in the statins-treated cells relative to that of the control cells (0.1%DMSO-treated cells) (Figure 4A).

We then administered statins in combination with MVA, FPP, or GGPP to investigate whether the inhibition of ERK1/2 and Akt activation in C6 glioma cells was due to the inhibitory action of statins on FPP or GGPP biosynthesis via their mechanism of action. Statins inhibited the activation of ERK1/2 and Akt, whereas in combination with GGPP, the activation levels of these signal transduction molecules were restored to the degree observed in control cells (0.1% DMSO-treated) (Figure 4B). These observations suggest that the inhibition of ERK1/2 and Akt activation in C6 glioma cells treated with statins was due to the inhibition of GGPP biosynthesis.

## Discussion

In the present study, we have demonstrated that statins inhibit C6 glioma cell proliferation. We have also found that statins induce apoptosis by activation of caspase-3 through inhibition of GGPP biosynthesis. It has been reported that statins inhibit prenylation of small G proteins by suppressing the production of GGPP [4,8]. Lovastatin is known to inhibit the mevalonic acid and MAPK pathways, thereby inducing apoptosis [9,10]. It has been reported that the mechanism of action is inhibition of GGPP biosynthesis [10,11]. These findings suggest that statins induce apoptosis by activation of caspase-3 through suppression of GGPP biosynthesis.

**Figure 5:**
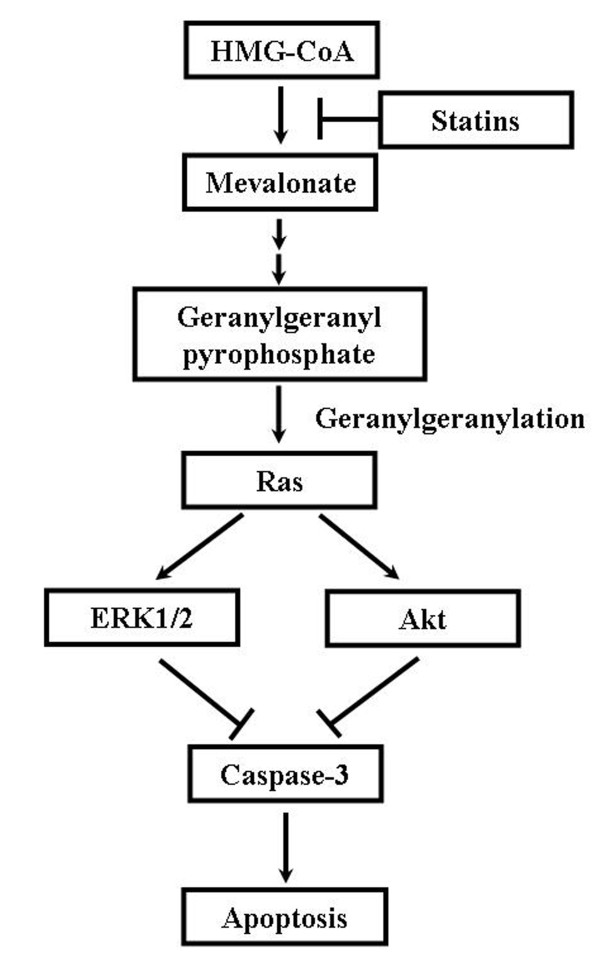
**Schematic representation of interacellular effects of statins in C6 glioma cells**.

GGPP is an important membrane-anchoring molecule of Ras protein. A shortage of GGPP facilitates dissociation of Ras from the inner surface of the membrane, and decreases the Ras-mediated growth signal, thereby inhibiting cellular proliferation [12,13]. Our results clearly demonstrate that statins induce a decrease in ERK1/2 and Akt activation of Ras downstream, but the activation of JNK1/2 was not altered. We previously reported that mevastatin induces a decrease in phosphorylated ERK [3]. We also demonstrated that fluvastatin and simvastatin decrease the activation of ERK1/2 Akt [4]. These findings are in agreement with the results of the present study and indicate that statins induce apoptosis via suppression of Ras/ERK and Ras/Akt pathways in our experimental model (Figure 5).

As described above, statins are known to affect the functions of Ras by inhibiting prenylation through the inhibition of GGPP synthesis; this enables localization of Ras at the plasma membrane [14,15]. Ras is involved in the activation of the MEK/ERK and PI3K/Akt pathways [14,16], suggesting the mechanism of action of statins.

The treatment of C6 glioma cells with 5 μM mevastatin, 5 μM fluvastatin or 10 μM simvastatin for 72 h in vitro inhibited GGPP synthesis. We also found that the treatment of C6 glioma cells with 2.5 μM mevastatin, 1 μM fluvastatin or 5 μM simvastatin for 72 h inhibited cell proliferation. The peak plasma concentrations of fluvastatin or simvastatin achieved with standard doses were ≤ 1 μM or 2.7 μM, respectively [17,18]. It has been reported that peak plasma concentration of fluvastatin achieved with high dose were ≤ 2 μM [19]. These findings indicate that 2 μM and 2.5 μM of fluvastatin and simvastatin, respectively, are within the peak plasma values of fluvastatin or simvastatin that are likely to be achieved in vivo. In addition, we found that 2.5 μM fluvastatin induced the apoptosis. Therefore, fluvastatin may be potentially useful as anti-cancer agents in the treatment of glioblastoma.

## Conclusion

In conclusion, these results provide evidence of the specific molecular pathways via which statins induce apoptosis by increasing the activation of caspase-3 through inhibition of Ras/ERK and Ras/Akt pathways. The findings indicate that statins may act more effectively on tumors that have spread on Ras-variable tumors. This further suggests that statins may be potentially useful as anti-cancer agents in the treatment of glioblastoma.

## Competing interests

The authors declare that they have no competing interests.

## Authors' contributions

MY and MT carried out cell viability assay, caspase-3 activity assay, statical analysis, and drafted the manuscript. TS, TI, MI, and YY carried out western bolotting analysis. TS, TI, and MI contributed to statistical analyses. SN designed the experiments and revised the manuscript. All authors read and approved the final manuscript.
